# Caution, “normal” BMI: health risks associated with potentially masked individual underweight—EPMA Position Paper 2021

**DOI:** 10.1007/s13167-021-00251-4

**Published:** 2021-08-17

**Authors:** Olga Golubnitschaja, Alena Liskova, Lenka Koklesova, Marek Samec, Kamil Biringer, Dietrich Büsselberg, Halina Podbielska, Anatolij A. Kunin, Maria E. Evsevyeva, Niva Shapira, Friedemann Paul, Carl Erb, Detlef E. Dietrich, Dieter Felbel, Alexander Karabatsiakis, Rostyslav Bubnov, Jiri Polivka, Jiri Polivka, Colin Birkenbihl, Holger Fröhlich, Martin Hofmann-Apitius, Peter Kubatka

**Affiliations:** 1 Predictive, Preventive and Personalised (3P) Medicine, Department of Radiation Oncology, University Hospital Bonn, Rheinische Friedrich-Wilhelms-Universität Bonn, 53127 Bonn, Germany; 2 Clinic of Obstetrics and Gynaecology, Jessenius Faculty of Medicine, Comenius University, in Bratislava, 03601 Martin, Slovakia; 3 Weill Cornell Medicine-Qatar, Education City, Qatar Foundation, 24144 Doha, Qatar; 4 Department of Biomedical Engineering, Faculty of Fundamental Problems of Technology, Wrocław University of Science and Technology, 50-370 Wrocław, Poland; 5 Departments of Maxillofacial Surgery and Hospital Dentistry, Voronezh N.N. Burdenko State Medical University, Voronezh, Russian Federation; 6 Stavropol State Medical University, Stavropol, Russian Federation; 7 Nutrition Department, Ashkelon Academic College, Ashkelon, Tel Aviv, Israel; 8 NeuroCure Clinical Research Centre, Experimental and Clinical Research Centre, Max Delbrueck Centre for Molecular Medicine and Charité Universitaetsmedizin Berlin, Berlin, Germany; 9 Private Institute of Applied Ophthalmology, Berlin, Germany; 10 European Depression Association, Brussels, Belgium; 11 AMEOS Clinical Centre for Psychiatry and Psychotherapy, 31135 Hildesheim, Germany; 12 Fachklinik Kinder und Jugendliche Psychiatrie, AMEOS Klinikum Hildesheim, Akademisches Lehrkrankenhaus für Pflege der FOM Hochschule Essen, Hildesheim, Germany; 13 Institute of Psychology, Department of Clinical Psychology II, University of Innsbruck, Innsbruck, Austria; 14 Ultrasound Department, Clinical Hospital “Pheophania”, Kyiv, Ukraine; 15 Zabolotny Institute of Microbiology and Virology, National Academy of Sciences of Ukraine, Kyiv, Ukraine; 16 Department of Neurology, Faculty of Medicine in Pilsen, Charles University and University Hospital Pilsen, Pilsen, Czech Republic; 17 Department of Histology and Embryology, Faculty of Medicine in Pilsen, Charles University, Staré Město, Czech Republic; 18 Biomedical Centre, Faculty of Medicine in Pilsen, Charles University, Staré Město, Czech Republic; 19 Department of Bioinformatics, Fraunhofer Institute for Algorithms and Scientific Computing (SCAI), Schloss Birlinghoven, 53757 Sankt Augustin, Germany; 20 Bonn-Aachen International Centre for IT, Rheinische Friedrich-Wilhelms-Universität Bonn, 53115 Bonn, Germany; 21 UCB Biosciences GmbH, Alfred-Nobel Str. 10, 40789 Monheim am Rhein, Germany; 22 Department of Medical Biology, Jessenius Faculty of Medicine, Comenius University in Bratislava, 03601 Martin, Slovakia

**Keywords:** Predictive preventive personalised medicine (3PM/PPPM), BMI deviation, Body weight, Anthropometrics, Well-being, Youth, Adults, Elderly, Overweight, Underweight, Nutrition, Deficits, Individualised patient profile, Communicable, Non-communicable disorders, COVID-19, Disease development, Manifestation, Progression, Pathology, Vasoconstriction, Endothelin-1, Flammer syndrome, Systemic ischemia, Hypoxic effects, ROS, Inflammation, Cardiovascular disease, Cancers, Neurology, Stroke, Neurodegeneration, Immune system, Wound healing, Reproductive dysfunction, Pregnancy, Artificial intelligence in medicine, Multi-parametric analysis, Big data management, Multi-level diagnostics, Body fluids, Modelling, Fat, Weight loss, Intentional, Unintentional, Biomarker panel, Molecular patterns, Metabolic pathways, Microbiome, Medical imaging, Healthcare, Sports medicine, Anorexia athletica, Population health, Innovative population Screening Programme, Health economy, Health policy

## Abstract

An increasing interest in a healthy lifestyle raises questions about optimal body weight. Evidently, it should be clearly discriminated between the standardised “normal” body weight and individually optimal weight. To this end, the basic principle of personalised medicine “one size does not fit all” has to be applied. Contextually, “normal” but e.g. borderline body mass index might be optimal for one person but apparently suboptimal for another one strongly depending on the individual genetic predisposition, geographic origin, cultural and nutritional habits and relevant lifestyle parameters—all included into comprehensive individual patient profile. Even if only slightly deviant, both overweight and underweight are acknowledged risk factors for a shifted metabolism which, if being not optimised, may strongly contribute to the development and progression of severe pathologies. Development of innovative screening programmes is essential to promote population health by application of health risks assessment, individualised patient profiling and multi-parametric analysis, further used for cost-effective targeted prevention and treatments tailored to the person. The following healthcare areas are considered to be potentially strongly benefiting from the above proposed measures: suboptimal health conditions, sports medicine, stress overload and associated complications, planned pregnancies, periodontal health and dentistry, sleep medicine, eye health and disorders, inflammatory disorders, healing and pain management, metabolic disorders, cardiovascular disease, cancers, psychiatric and neurologic disorders, stroke of known and unknown aetiology, improved individual and population outcomes under pandemic conditions such as COVID-19. In a long-term way, a significantly improved healthcare economy is one of benefits of the proposed paradigm shift from reactive to Predictive, Preventive and Personalised Medicine (PPPM/3PM). A tight collaboration between all stakeholders including scientific community, healthcare givers, patient organisations, policy-makers and educators is essential for the smooth implementation of 3PM concepts in daily practice.

## Introduction

### Overweight and obesity

Overweight parameters are as follows: for females, body mass index (BMI) 25–30 and for males, BMI 26–30.

Obesity parameters are as follows: class I—BMI 30–35; class II—BMI 35–40; class III—BMI > 40.

According to the WHO, in 2016, 50 million girls and 74 million boys around the globe were registered as being obese. In Europe, problems of overweight are rapidly increasing in most of the member states demonstrating about 52% of the EU population aged > 18 years. Moreover, whereas in 2008, 25% of children aged 6–9 years were overweight or obese in Europe, in 2010, their number reached already 33%. Considering severe health risks linked to overweight in the population, the European Commission has launched a European platform with more than 300 initiatives to promote anti-overweight measures in the EU [1]. The aim is to combat severe physical and mental health adverse effects of overweight and obesity as both conditions are strongly associated with changes on the biological level, including increased inflammatory signalling, elevated levels of highly aggressive reactive oxygen species (ROS) and altered endocrine stress hormone signalling that synergistically may cause enormous burden of allostatic load lowering the resilience and coping ability of the body e.g. under chronic and traumatic stress.

### Underweight

Underweight parameters are as follows: grade I: females, BMI < 19, males, BMI < 20; grade 2: BMI ≤ 17; grade 3: BMI ≤ 16.

In 2016, 75 million girls and 117 million boys globally were registered as moderately or severely underweight. Exemplified by French girls, against the year 1998, in 2006, the likelihood increased by 41% for them to be abnormally thin or even underweight.

**Fig. 1:**
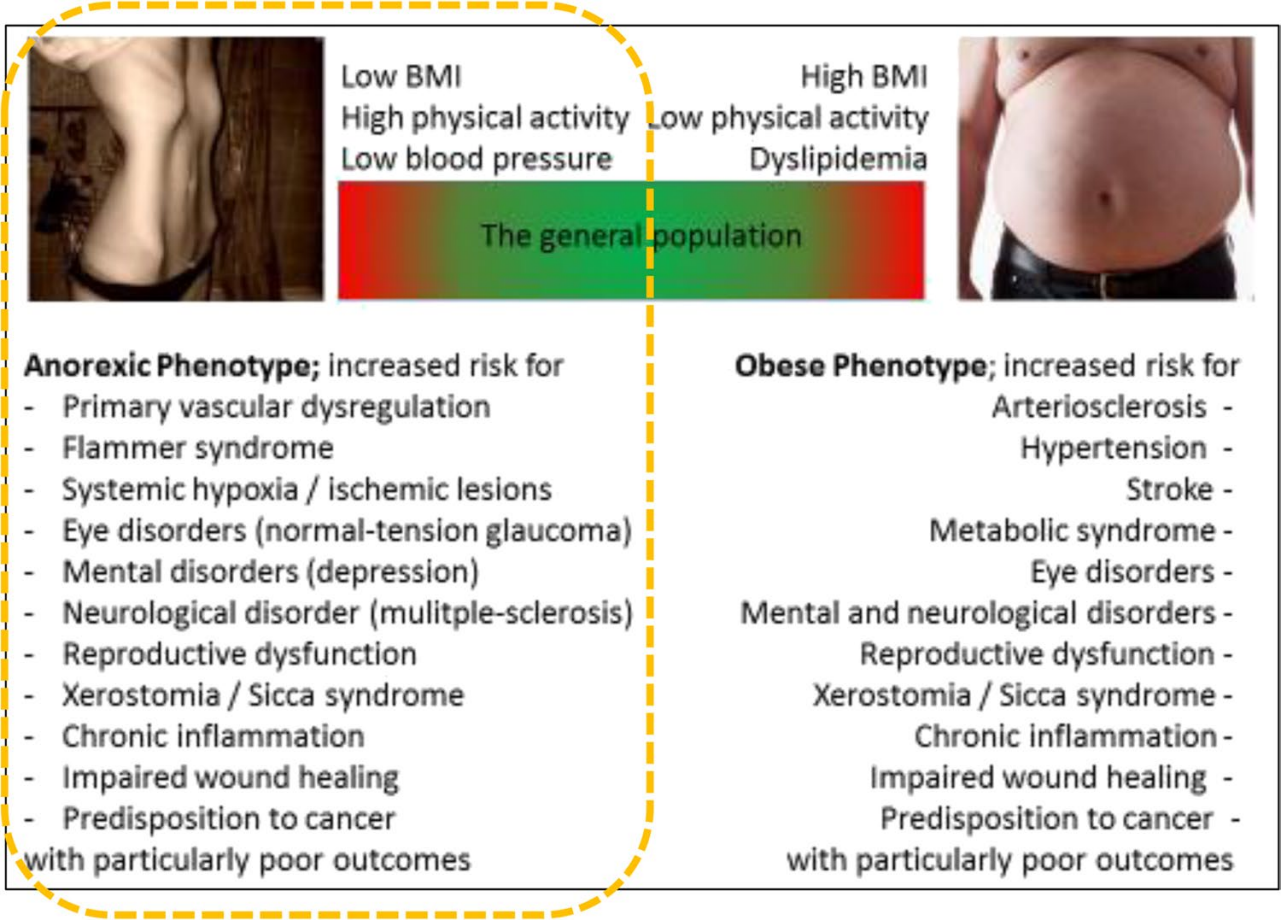
Anorexic versus obese phenotype: The paradox of the similarity of health risks; the figure is adapted from [4]

Recent studies demonstrate that thinness is an overlooked phenomenon in terms of causality, health risks and associated pathologies which are much less explored compared to those associated with overweight and obesity [2, 3]. Increased risks of syndromes and pathologies linked to both phenotypes, namely obese versus anorexic ones, are schematically presented in Fig. 1. Corresponding expertise is strongly multi-professional comprising cardiovascular, ophthalmologic, neurologic, psychologic, psychiatric, gynaecologic, urologic, oncologic, otorhinolaryngologic and dental, amongst others [4].

Consequently, increasing interest in a healthy lifestyle raises questions about an optimal body weight. Evidently, a *statistically normal weight* does not mean *individually optimal weight* following the basic principle of personalised medicine “one size does not fit all”. Contextually, “normal” borderline BMI values such as 19–20 kg/m^2^ might be optimal for one person but apparently suboptimal for another one strongly depending on the individual genetic predisposition, geographic origin, cultural and nutritional habits and other lifestyle parameters.

Within the standardised “normal” BMI range, which value is the personal optimum? In order to better understand the challenge of answering the question, this is to exemplify the difference which corresponds to minimal versus maximal value of the “normal” range:

A. for the female height of 160 cm, according “normal” body weight may range between 47.5 and 64 kg with the difference of 16.5 kg (35%)
B. for the male height of 190 cm, according “normal” body weight may range between 66.5 and 90.5 kg with the difference of 24 kg (36%).

To this end, 35–36% of the body weight difference, if considered for the same person, would reflect significant shifts in the body composition with corresponding alterations in the tissue, cellular and molecular set-up such as muscles to the water and fat ratio, systems efficiency, free-radical production and activity of inflammatory signalling cascades, turnover and quality of metabolic processes and energy supply.

The current article highlights potential health risks associated specifically with the low body weight as well as intentional and unintentional weight loss to be considered for prediction of individual risks, targeted prevention of health adverse effects and health-promoting recommendations tailored to the person in the framework of 3P medicine.

## Association of low and high BMI with overall mortality risks—generalised versus individualised risk presentation

In 2018, the association of BMI with overall and cause-specific mortality has been analysed within a population-based cohort of over 3.6 million adults in the UK [5]. In general, a “J”-shape association was demonstrated between BMI and overall mortality. However, the range of lowest risks differed between cause-specific mortality. To this end, the generalised lowest risks associated with cancers and cardiovascular diseases were demonstrated for the range 21–25 kg/m^2^, whereas lowest risks for mental, behavioural, neurological and accidental causes were associated with the range 24–27 kg/m^2^. Further to this, disease-dependent mortality demonstrated highly individual shape of risks associated with the BMI:

- “J”-shape (higher risks at high BMI) was characteristic for cardiovascular diseases demonstrating risks associated with increasing BMI
- A mirrored “J” shape (higher risks at low BMI) was demonstrated for neurological, mental and behavioural causes demonstrating an inverse association with increasing BMI
- Respiratory disorders demonstrated “U” risks associated with low and high BMI
- For mortality from self-harm, an inverse linear association “\” was demonstrated: the risks were significantly decreased by increasing BMI.

Importantly, an association between BMI and mortality risks was stronger at younger ages.

Notably, increased mortality rates associated with a low BMI were demonstrated as being characteristic e.g. for neurological dementia and mental/behavioural causes of death, whereas high BMI is strongly associated with hypertensive heart disease as the cause of death.

**Fig. 2:**
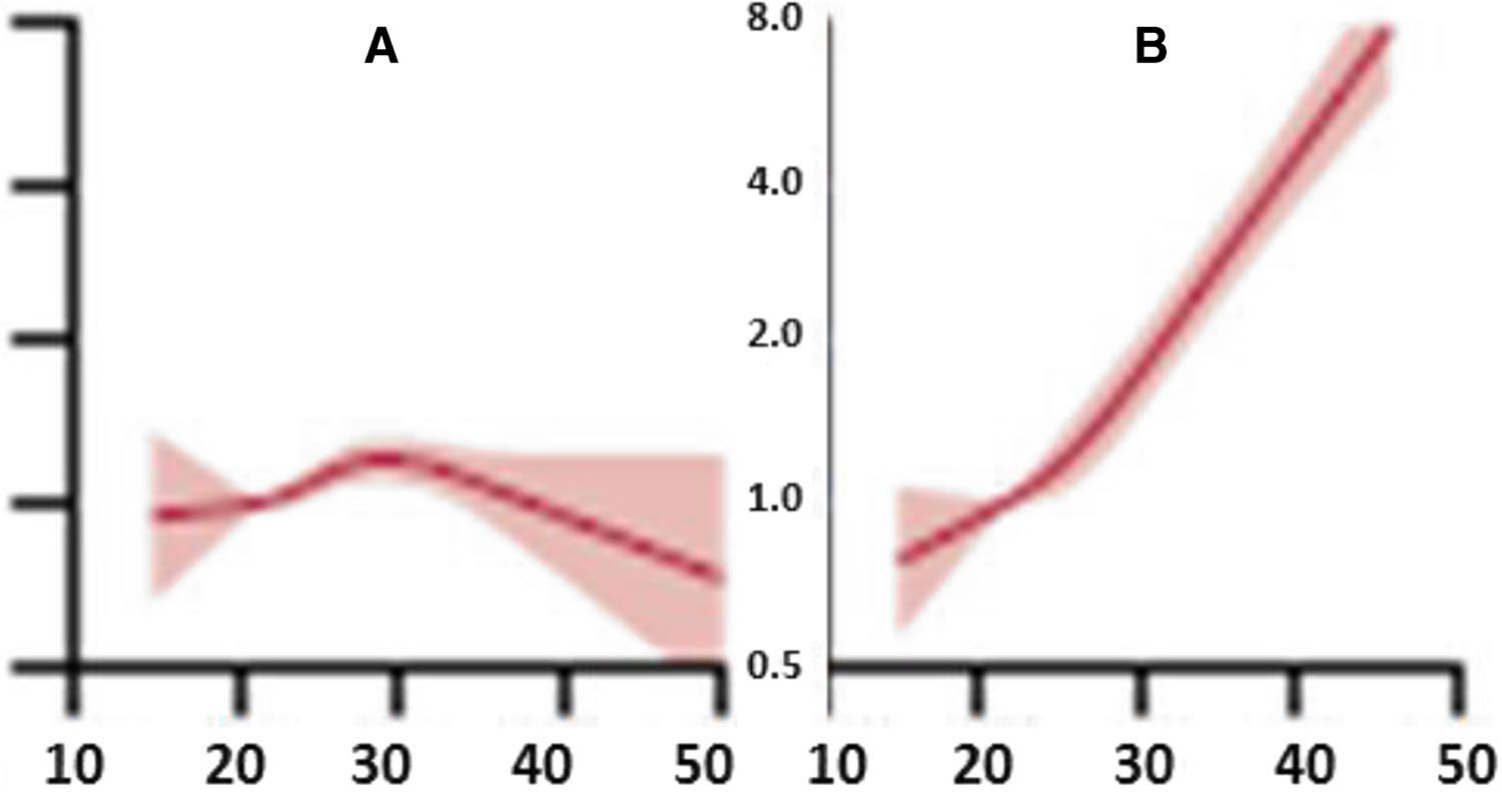
A population-based cohort study by Bhaskaran et al. demonstrated the association between BMI and cause-specific mortality, exemplified here for never-smokers diagnosed with **A** prostate cancer versus **B** uterus cancer; the horizontal axis indicates BMI (kg/m^2^) and the vertical axis indicates hazard ratio (95% confidence interval); the image is adapted from [5]

However, it is crucial to emphasise that BMI is a highly individual parameter to be considered in the context of many other factors as exemplified for prostate cancer (PCa) in Fig. 2.

**Fig. 3:**
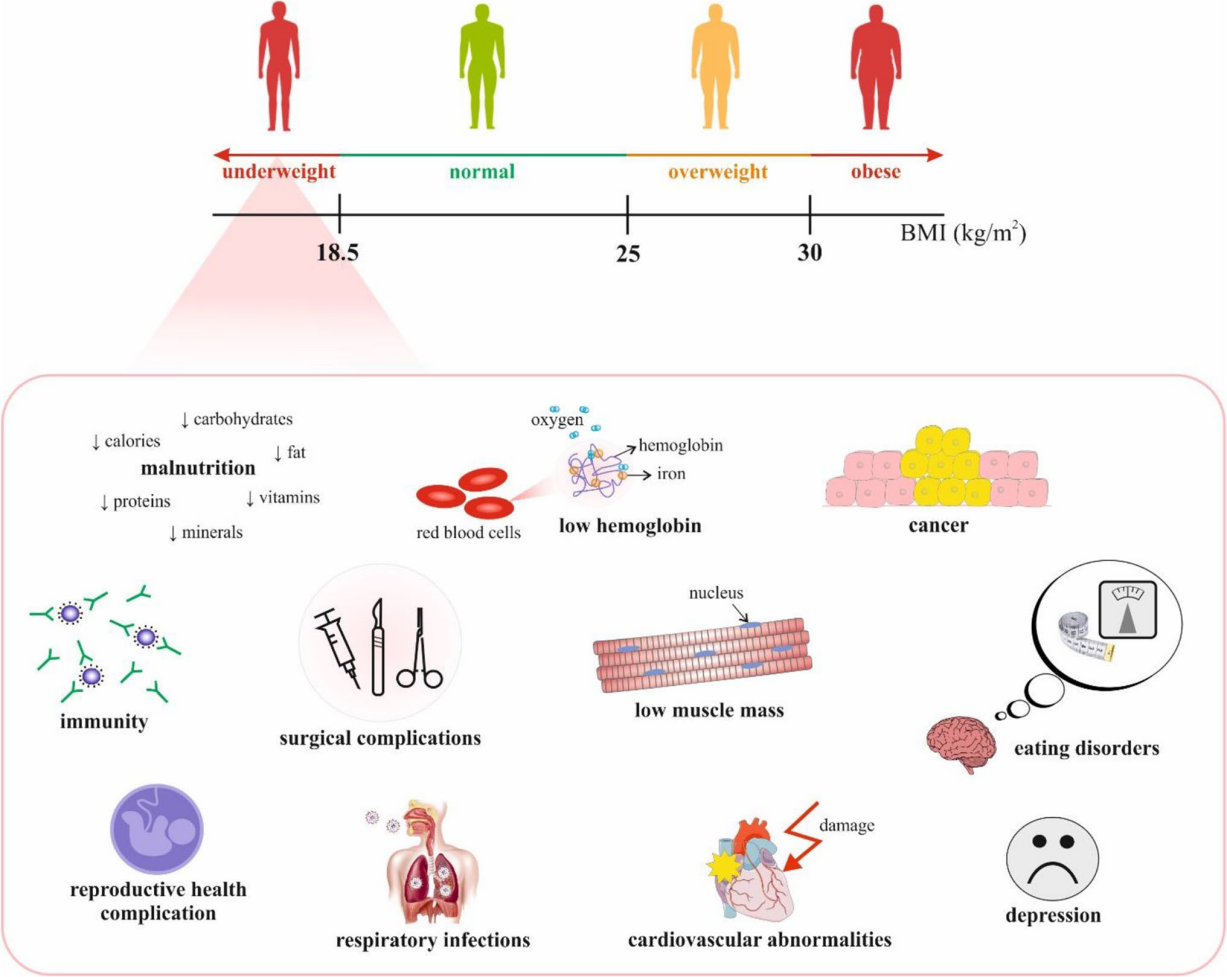
Health risks potentially associated with low body weight; *Explanatory notes:* BMI classification: *underweight*—BMI < 18.5 kg/m^2^; *normal weight*—BMI = 18.5 to 25 kg/m^2^; *overweight*—BMI ≥ 25 to < 30 kg/m^2^; *obese*—BMI ≥ 30 to < 35 kg/m^2^; and *severely obese*—BMI ≥ 35 kg/m^2^ [40, 41]. Selected suggested mechanisms behind increased risk of specific health complication associated with underweight include the following: abnormal nutritional status, low body fat (e.g. anorexia athletica) or low muscle mass, muscular atrophy [42], cardiovascular abnormalities, valvular dysfunction, compromised immunity [43]; *cancer*—particularly poor outcomes of some cancers, potentially decreased tolerability/effectiveness of cancer treatment e.g. due to lower haemoglobin and albumin resulting from abnormal nutritional status, cachexia, impaired anti-tumour immunity [44], loss of muscle fat mass, sarcopenia [45], increased risk of several cancer types and metastatic disease [6, 46, 47]; *impaired healing and increased post-surgical complications*—abnormal nutritional status, insufficient energy supply, shifted metabolic pathways and microbiome alterations [4, 24, 48],  potentially low preoperative haemoglobin [49, 50]; *reproductive dysfunction*—disruption of hypothalamic-pituitary–gonadal axis leading to hypothalamic anovulation [51], ovulatory dysfunction [52], negative effects on IVF parameters [53–55]; *compromised immunity*—abnormal nutritional status, lymphopenia [56]; *respiratory infections including COVID-19*—malnutrition [57], coexisting chronic conditions [58], immuno-suppression as a result of malnutrition [59]; *eating disorders (anorexia nervosa)*—negative effects on overall and reproductive health [60]; neurological disorders such as young stroke [12] and abnormal pain sensitivity / perception [4, 7]; *abnormal sleep patterns *[7, 11, 15]* and depression* [61]; *primary vascular dysregulation*—abnormal nutrition, low energy supply, Flammer syndrome, high Endothelin-1 level in blood plasma, increased stress sensitivity, amongst others [4, 9, 15, 17, 62]; Sicca syndrome with severe complications [11, 13, 15].

To this end, being a multifactorial disease PCa results from a highly comprehensive interplay between exogenous and endogenous risk factors as clearly illustrated by the complex association between the body mass index (BMI) and PCa-related mortality (Fig. 3). Whereas uterus cancer and related mortality are positively associated with overweight and obesity, for the PCa, this association is not that clear. Rather, in contrast, there is a mild decrease in mortality rates for obese PCa patients compared to those with normal and low BMI [6]. Contextually, individualised profiling is instrumental for data interpretation such as BMI.

## Focus on adolescents: predisposition to intentional anorexic phenotype may appear early in life

Unmet needs in the healthcare of young populations in a long-term way cause epidemics of non-communicable disorders. Currently, an unprecedented decrease in the average age of clinically manifested chronic disorders is observed being characteristic for the early twenty-first century. To this end, teenagers are more and more frequently diagnosed with type 2 diabetes mellitus type and depression; there is an increasing incidence of mood disorders and suicide in youth, alarming statistics of vascular stiffness in young populations, “young” strokes (> 50 years of age) with unknown aetiology, reproductive dysfunction, aggressive metastasising cancers in the 2nd and 3rd life-decades with particularly poor outcomes, increasing prevalence of preventable eye disorders, inflammatory processes and allergic reactions and autoimmunity, and respiratory disorders, amongst others [4].

A paradox is that specifically in adolescents, the adverse health effects of suboptimal health conditions are reversible in most cases. This unique capacity is, however, not adequately utilised by current concepts of healthcare: still, the clinical manifestation of the disease is the acknowledged indicator for conventional medical services. However, the majority of non-communicable disorders carry a chronic character by progressing over a couple of years from a reversible suboptimal health condition to irreversible pathology with collateral complications. The timeframe between both conditions is the operational area for predictive diagnosis and identification of persons at risk by innovative screening programmes followed by the most cost-effective personalised treatment possible, namely primary prevention tailored to the person.

Specifically, in adolescents, physical and mental health linked to body shape plays the central role for major aspects such as successful career development, sexual life and long-term partnering. Suboptimal health conditions make them vulnerable to associated pathologies early in life. For example, a sedentary lifestyle leads to physical inactivity and weight gain with potentially cascading pathologies. To this end, German statistics demonstrate that from 100 teenagers aged 12–16 years, 11 are overweight and 9 obese. Similar numbers are recorded for many other industrial countries [4]. However, inadequate discussion on the matter gave rise to the opposed extreme situation which is not a less alarming trend: In Germany, about 50% of female teenagers and 25% of male teenagers with physiologically normal body shape do believe to be overweight undergoing unsupervised dieting early in life. “Disordered eating” frequently leads to the clinical manifestation of eating and mental disorders as well as substance abuse in adolescence.

Amongst reported eating disorders, the absolute majority of cases are represented by anorexia nervosa and bulimia, wherefrom a great portion of bulimia cases originates from treated anorexia nervosa. The pubertal changes usually increase awareness of an individual body shape, when teenagers consider their bodies with enormous self-criticism. To this end, if anybody expresses a negative opinion about their shape particularities, this might negatively impact individual attitude in a long-term manner. Contextually, the most frequently discussed parameter is certainly the body weight and thinness as the symbol of beauty leading to the strong dieting intentional weight loss—the conceptual beginning of “disordered eating”. Frequently, the familiar environment shapes beauty symbol by thinness. Experienced psychiatrists report specific behavioural patterns associated with the following:

- dieting mothers: if the mother is permanently talking about or undergoes dieting frequently, her daughter(s) follow this behavioural pattern; even after the mother stops dieting, children often continues uncontrolled starvation until their condition is diagnosed as an “eating disorder”
- children of academics: for example, children of teachers are particularly systematic and perfectionistic in dieting and developing anorexic conditions
- overprotected children: hyperactive parents strongly promote their initiatives focused on the children protection that significantly suppresses an adequate development of own interests, activities and competencies by children, who simply follow the behavioural patterns of their parents
- “stiff” families which are highly conservative following traditions; any change is considered to be damaging the family and may lead to disordered behaviour of affected children including eating and mental disorders.
- “conflict-mitigating” behavioural pattern in the family, when parents are avoiding any opinion difference in the family as potentially “destabilising” that may lead to eating and mental disorders of teenagers.

Notably, particularly affected are usually teenagers with a meticulous personality and the tendency to perfectionism. Further to this, mood disorders such as depression go hand-in-hand with eating disorders as detailed in [4].

Finally, adverse childhood experiences may play a role as a vulnerability factor for eating disorders. Childhood maltreatment (CM) comprises any kind of experience of physical, sexual or emotional abuse as well as physical and emotional neglect. According to the World Health Organization, the worldwide prevalence is only estimated as precise numbers for any country are missing. However, the WHO provided data claiming that about 25% of all adults were exposed to physical abuse. For sexual abuse, the prevalence is about 20% for women and 8% for men, with about 13.5% of all children expected to be exposed to at least one experience of sexual abuse. Emotional abuse and physical abuse are the most present forms of CM and adverse childhood experiences (ACE) are not single events but have to be considered chronic and repetitive stressors and therefore limit the resilience and coping capability of affected subjects in a dose–response relationship. These findings are of epidemiological, psychosocial and medical interest as ACE and CM are reported in literature to significantly contribute to the aetiology and manifestation of eating disorders on the one side and on the other side, ACE and CM are hardly considered in these clinical cohorts in the context of personalised diagnostic approach, treatment modalities and relapse prevention.

## Abnormal stress reactions as a general risk of disease development, manifestation and progression

Flammer syndrome phenotype demonstrating characteristic low body weight and abnormal reactions towards any kind of physical (e.g. cold provocation) and mental stress in affected individuals has been described [7, 8] as being strongly associated with an increased incidence of communicable and non-communicable disorders in this cohort [6, 9–15]. The most prominent contributing factors and corresponding pathomechanisms are summarised below.

### Voice disturbances under stress overload and associated observations

A recently performed clinical study suggested voice disturbances as an indicator of an individual’s stress overload: the authors hypothesised that the voice perturbation in young (19–22 years old medicine students) and otherwise healthy individuals might be positively associated with vascular dysregulation and dry mouth syndrome[15]. Indeed, the study revealed voice perturbations under the stress overload as a potentially useful biomarker to identify individuals in suboptimal health conditions potentially vulnerable to related pathologies developed later on in life. To this end, low BMI (≤ 20) and symptoms of dry mouth syndrome and disturbed microcirculation were positively associated with voice perturbations under stress overload. Multivariate analysis applied to the same cohort, further, demonstrated a positive association between the following:

- perfectionistic altitude and sleep deficits (*P* < 0.01) as well as Sicca syndrome (*P* < 0.01)
- increased pain perception and impaired wound healing (*P* = 0.04)
- stroke cases in the family and frequent migraine with complications such as aura (*P* = 0.004) as well as evidently shifted circadian rhythms (*P* = 0.04)—all well-acknowledged risk factors in the proposed aetiology of the “young” stoke [12].

The authors recommended developing an automated analysis of voice records by application of artificial intelligence with the potential to derive digital biomarkers. Further to this, it has been concluded that predictive machine learning models might be useful for detecting a suboptimal health condition based on voice records followed by the detailed patient stratification utilising disease-specific cell-free nucleic acids amongst other multi-omic technologies [16]. Application of the cost-effective targeted prevention as long as health damage remains reversible was recommended based on the individualised patient profiling [15].

### Lasting vasospastic reactions

Under stress conditions, lasting vasospasm is caused by high blood plasma level of the vasoconstrictor endothelin-1 (ET-1) strongly associated with systemic ischemic-hypoxic effects. In turn, ET-1 axes regulate a myriad of processes involved in a modulation of physical and mental well-being, female and male health, senses, pain, stress reactions, drug sensitivity and healing processes, amongst others. Imbalanced ET-1 overproduction modulates individual outcomes in the development and progression of communicable infections such as COVID-19 and non-communicable disorders such as metabolic impairments with cascading complications, ageing and related pathologies, cardiovascular diseases, neurodegenerative pathologies, and aggressive malignancies [17].

### Psycho-immunology

Imbalanced stress reactions in a long-term manner may cause mood disorders such as depression which is strongly associated with compromised functionality and auto-regulation of the immune system [18]. Chronic activation of the hypothalamic–pituitary–adrenal (HPA) axis in the stress response overload evidently impairs the immune response leading to the development and progression of related diseases such as cardiovascular and cerebrovascular conditions, metabolic and autoimmune disorders and even cancers. Per evidence, stressors and depression are associated with the accumulation of somatic mutations and instability of genomic and mitochondrial DNA on the one hand and, on the other hand, with decreased cytotoxic T cell and natural-killer-cell activities that affect the immune surveillance of tumours.

### Sleep deprivation

Caused by abnormal stress reactions, sleep shortage and disturbances are per evidence the risk factors and facilitators of a broad spectrum of disorders, including mood disorders, stroke, chronic inflammation, immune defence insufficiency and cancer. The reciprocal interrelationship between the abnormal stress, sleep quality and individual outcomes became evident under extreme conditions such as the COVID-19 pandemic [19].

### Mitochondrial dysfunction

Stress overload is a well-acknowledged risk factor of mitochondrial injury. In turn, mitochondriopathies play a key role in the aetiopathology of multifactorial diseases exhibiting a “vicious circle” characteristic for multi-organ damage and failure in the long run. Uncontrolled release of reactive oxygen species, diminished energy production in terms of adenosine triphosphate (ATP) resources and extensive damage to the life important biomolecules—synergistically present the generalised pathomechanisms of cascading pathologies frequently developed in a reciprocal manner [20, 21]. Additionally, not only changes in the quality of mitochondrial bio-energetic output might contribute to the pathophysiology of stress-associated disorders, but also changes in the regulation of mitochondrial density inside the cells, further controlled by fission-and-fusion dynamics as well as mitophagy in stressed cellular systems. As a result, the quantitative bio-energetic output of mitochondria should also be normalised to the mitochondrial density inside cells of interest to correctly characterise the nature of the observation (mitochondrial dysfunction vs. impairment) when testing mitochondrial bioenergetics and the integrity of mitochondrial and genomic DNA in physical and mental health and disease conditions.

### Systemic inflammation

Under imbalanced stress conditions, there are evident cumulative systemic effects leading to chronic inflammation including but not restricted to the lasting vasospastic reactions with systemic ischemic-hypoxic effects and involvement of ET-1 and neuro-immune axes, mitochondrial impairments and increased infection susceptibility with poor outcomes such as caused by the cytokine storm reported for COVID-19-infected individuals [19, 22]. Chronic systemic inflammation is key risk factor in several non-communicable diseases such as cancers [23].

### Impaired healing

Delayed and impaired healing is a multifactorial condition frequently associated with both abnormal body weight and stress overload [4, 24, 25]. In turn, impaired healing is indicative and predictive for a development and progression of many associated pathologies such as an aggressive metastatic disease, which is considered a “non-healing wound” [26]. Cellular responses to inflammation in both wound healing and metastasis are similar being tightly regulated by crosstalk with the surrounding microenvironment. Contextually, targeting canonical responses to inflammation is a novel strategy to prevent impaired healing and metastatic disease.

## What is known about adverse effects of both intentional and unintentional weight loss within the “normal” BMI range?

### Unintentional weight loss—prominent examples

Unintentional weight loss (UWL) comprises a diagnostic challenge for clinicians. Indeed, the main etiologic groups of UWL are represented by malignancies, non-malignant organic disorders and psychiatric disorders. To this end, unexplained UWL accounts for 11–28% of cases. Further to this, UWL is quite prevalent in the elderly and is considered a distinct clinical entity [27]. UWL is characterised as a more than 5% reduction in body weight within 6 to 12 months [28]. Rapid and pronounced UWL indicates potentially underlying disease. For example, extensive cytokine release resulting from chronic or acute diseases may induce anorexia nervosa development, stimulate lipolysis, muscle protein breakdown and/or nitrogen loss [29].

#### Malnutrition

BMI is a common measure of nutritional status in adults [30]. There is a close association between UWL and malnutrition. Indeed, malnutrition can be defined as (a) BMI < 18.5 kg/m^2^ or (b) UWL > 10% of initial body weight irrespective of time or > 5% during last 3 months combined with either BMI < 20 kg/m^2^ if < 70 years of age, or BMI < 22 kg/m^2^ if older than 70 years or fat-free mass index corrected for body size (FFMI) < 15 in women and < 17 kg/m^2^ in men. Indeed, low BMI is not always associated with malnutrition while individuals with increasing BMI may have decreasing FFMI [31]. Malnutrition can result in various complications such as delayed and impaired wound healing, an increase in postoperative morbidity and prolonged hospitalisation. To this end, early screening for patients at risk might lead to improved diagnostics and treatment approaches [32].

#### Elderly

UWL is relatively prevalent in the elderly that is challenging. Indeed, 15–20% of adults aged ≥ 65 years demonstrate UWL with the prevalence higher in dwelling elders and nursing home residents [27]. Rapid UWL in the elderly indicates associated diseases and accelerate loss of muscles mass compared to physiological ageing [29]. UWL is strongly associated with increased morbidity and mortality rates in older populations [28]. Notably, malignancies, non-malignant organic disorders, non-malignant gastrointestinal disease, psychiatric and/or psychosocial disorders are the most common causes of the UWL in the elderly [27, 28] with aetiology of the UWL similar to each other being also age-independent [27]. Further to this, Gaddey et al. reported on the non-malignant diseases as a more common cause of UWL in the older populations than malignancies. Social factors also contribute to the UWL in the elderly. Importantly, UWL in the elderly can result in a functional decline in the activities of living and increased in-hospital morbidity, risk of hip fractures in women, and overall mortality. Moreover, cachexia described as a loss of muscle mass without loss of fat leads to infections, pressure ulcers and decreased response to treatments [28]. Moreover, low body weight in the elderly can result from decreased taste and smell as well as lack of appetite, due to reduced physical activity, drug interactions and dental complications [29]. Finally, low BMI has suggested a stronger predictor of premature death when compared with high BMI in older people [33].

#### Cancer

UWL is an important indicator of malnutrition among surgical cancer patients; the awareness of UWL as an indicator of malnutrition needs to be evaluated during cancer management, even if an individual BMI is within the normal range [32]. UWL commonly occurs to patients with locally advanced cervical cancer (LACC) receiving chemo-radiation that significantly affects individual outcomes. Although being a relatively common phenomenon, the weight loss issue in the LACC patient cohort is under-estimated. Consequently, the focus on low body weight is essential to improve LACC outcomes [34]. Significant body weight reduction during radiation treatment is associated with poor survival, for example, for patients with nasopharyngeal cancer; this risk factor is more prominent in the initially low body weight group of patients [30]. Similarly, UWL is highly problematic also for patients with the gastrointestinal cancer negatively impacting treatment efficacy and survival rates. Also, patients with upper gastrointestinal cancer and BMI < 20 kg/m^2^ have to be precisely evaluated for optimal weight management interventions, since they exert the highest mortality rates. Experts recommend dedicating a lot of attention to optimise body weight management in the overall cancer care and improved individual outcomes [35].

### Intentional weight loss raises questions

Over several past decades, clinical intervention programmes for intentional weight loss have been developed. Doubtlessly, for obese sub-populations, those programmes are the real hope for returning to a normal life. However, how much help versus potential harm can occur to a spectrum of individuals intending weight loss, if stratified by age, gender, cultural traditions, geographic particularities, individual genetic predisposition, lifestyle and professional occupation, amongst others? This issue is detailed in the subsequently presented sections. In general, body fat is suggested to be predicted from BMI considering both age and gender [36]. Per evidence, men lose more weight compared to women when utilising similar weight loss interventions, potentially due to higher initial body weight [37]. Further to this, men and women respond differently to rapid weight loss: men tend to benefit more than women from the metabolic outcomes. Men demonstrate more pronounced reductions in metabolic syndrome *Z*‐score, C‐peptide, fat mass and heart rate, while women exert larger reductions in HDL cholesterol, hip circumference, bone mineral content (BMC), FFMI and pulse pressure. To this end, declines in HDL cholesterol, BMC and lean mass were demonstrated to be necessarily health supportive in a long‐term way [38].

## Systemic effects and health risks associated with low body weight

The association between obesity and adverse health effects is widely recognised. In contrast, health risks associated with low body weight is much less explored. Certainly, being underweight increases mortality and reduces life expectancy [39]. Figure 3 highlights selected negative systemic effects of low BMI and underweight.

## Cardiovascular diseases

The association between cardiovascular disease and overweight is mediated by “traditional” risk factors such as hypertension and diabetes [63]. Although, much less is known about cardiovascular risks in low body weight individuals and despite significantly lower prevalence of the “traditional” risk factors in this subpopulation, per evidence, underweight is associated with more severe and extensive coronary disease compared with the normal weight or even obesity [64]. Further to this, lean body mass has been demonstrated as the predominant anthropometric risk factor of arterial fibrillation (AF). In contrast, any of the measures related to obesity have not been proved as an independent risk factor of the AF.

A cross-sectional study revealed a 19.7% greater risk of cardiovascular diseases of the underweight population compared with the normal-weight controls, specifically in the age category below 60. The association with the low body weight was found to be highly significant for the stroke risks [42]. To this end, young stroke of unclear aetiology has been hypothetically associated with the symptoms signs characteristic for individuals with the FS phenotype such as low body weight, abnormal stress reaction and vascular dysregulation [12].

Increased risk of cardiovascular diseases in the underweight sub-populations may be related to various factors, such as accelerated vascular ageing, sarcopenia, nutritional status, low body muscle mass and “metabolically obese underweight”, due to increased visceral fat (see the “Diagnostics of suboptimal low body weight” section) and metabolic abnormalities [42]. Many studies associate excessive mortality with confounding by cachexia, also known as unintentional weight loss, fatigue and muscular atrophy that is related to chronic diseases. Notably, Bucholz et al. demonstrated underweight as a risk factor of increased mortality in patients diagnosed with acute myocardial infarction (AMI) even independently of factors associated with cachexia such as comorbidities, frailty measures and laboratory markers of nutritional status. Higher mortality in the underweight patients’ cohort diagnosed with the AMI can be explained by an increased vulnerability to adverse health effects linked to additional weight loss, due to hospitalisation and increased infection susceptibility with complications [64]. Moreover, the Ohsaki study in Japan demonstrated a substantial association between underweight, haemorrhagic stroke and ischemic heart disease mortality, potentially due to the weakness of underweight patients (weak to survive). Further to this, being underweight was associated with an increased risk of cardiovascular abnormalities, valvular dysfunction, reduced ventricular mass, cardiac myofibril damage and compromised immunity [43]. Moreover, risks of total mortality, cardiovascular mortality and hospitalisation were the highest in the underweight chronic heart failure patients, while risks of cardiovascular mortality and hospitalisation were the lowest in overweight patients [65]. These results support the “obesity paradox” described as a better prognosis in overweight and mildly obese cardiovascular patients compared with lean cardiovascular-diseased counterparts [66–68]. Further to this, in the young population, vascular stiffness has been demonstrated to be a more frequent phenomenon in otherwise healthy but low-weight individuals compared to their overweight counterparts (unpublished data of project-dedicated EPMA expert group; publication in preparation). The proposed mechanism includes abnormal stress reactions, chronically high levels of the endothelin-1 in blood plasma and accelerated vascular ageing [17]. The EPMA expert group emphasises the need to differentiate between specific phenotypes in low-weight young populations; one of the stratification factors is connective tissue dysfunction. This dysfunction is clinically manifested by a whole set of external stigmas, amongst which the most frequent and accessible sign might be hypermobility of the joints further described in the “Reproductive dysfunction and pregnancy” section. The association between the connective tissue dysfunction and specific organ alterations such as vegetative vascular dystonia, heart valve prolapses, cerebral artery malformations and ruptures and abnormally located left ventricular chords have been described [69]. Also, some forms of an angiopathy based on the systemic endothelin-1 overproduction and increased vascular stiffness, which molecular mechanisms differ completely from usually observed vascular ageing, might be specific for this phenotype [65–68, 70]. Consequently, the EPMA expert group strongly recommends a detailed stratification of the affected individuals as being highly relevant for an accurate predictive diagnostics, targeted prevention and treatments tailored to the person.

## Young stroke of unclear aetiology

Stroke is one of the most devastating disorders globally demonstrating 1-month case-fatality rates ranging from 13 to 35%. The majority of cases appear in the advanced age population groups demonstrating well-acknowledged risk factors such as sedentary lifestyle, smoking, overweight, hypertension and abnormal sleep patterns. However, alarming statistics are provided by more recent studies demonstrating a persistently increasing stroke incidence in adolescents and young adults: “young” stroke (< 50 years of age) is a multifactorial disease frequently described as being of unclear aetiology [12]. An example is provided below.

### Stroke mimics and young stroke—case report

A 24-year-old female was presented as a stroke patient. She exhibited right-sided hemiparesis—moderate at the upper extremity, mild in the lower extremity and with mild Brocca’s aphasia. Multimodal CT was normal. A severe headache gradually developed, and hemiparesis and aphasia resolved. MRI ruled out stroke. The diagnosis was migraine with aura presented as a stroke mimic. In medical history, the patient suffered from headache in stressful situations and perimenstrual headache, overactive bladder, stress urinary incontinence, sleep disorder—falling asleep and insomnia, cold intolerance, excessive sweating, unpleasant perceived joint hypermobility, neck and low back pain and stress eating disorder accentuated during the university exam period. Clinical findings are as follows: weight 45 kg, height 171 cm, BMI ˂16. Blood pressure in sitting position 102/60, asthenic figure, joint and spine hypermobility, acrocyanosis and acral hyperhidrosis, stress skin stains and faltering stress speech.

In summary, the patient was finally diagnosed with migraine with aura presented as a stroke-like episode (stroke mimics). Patient was evidently underweight and demonstrated strongly pronounced symptoms and signs of Flammer syndrome such as low BMI, low blood pressure, disturbed microcirculation, dizziness, abnormal stress reactions, altered sense regulation and sleep patterns. Implemented treatment procedure comprised prophylactic migraine treatment with cinnarizinum, and magnesium therapy. Personalised approach and counselling were applied to maximise the treatment benefit. Partial reduction of symptoms occurred within follow-up of 22 months. She finished the university and started working as a trainee lawyer. The patient was included in the grant project of University Hospital Pilsen and Faculty of Medicine in Pilsen, Charles University, for the years 2021–2025 of detailed diagnostics, treatment and prevention of young stroke with potentially severe socio-economic consequences.

### Recommendations of the EPMA expert group

Less known risks specific for young populations and relevant for “young” stroke of unclear aetiology have to be explored such as low BMI, stress and ROS overload biomarkers and mitochondrial dysfunction, primary vascular dysregulation, endothelin-1 overproduction, vascular stiffness, systemic ischemic-hypoxic effects, low-grade inflammation and altered disease-specific molecular patterns in body fluids as well as their association with modifiable risk factors such as lifestyle, nutritional habits and supplements [15–17, 20, 21, 71]. Corresponding risk-mitigating measures are recommended for targeted prevention including innovative screening programmes by application of specialised questionnaires and biomarker panels. Educational programmes is an essential pillar in the prevention to be adapted to the target audiences such as children, adolescents and young adults [12].

## Pain of unclear aetiology—particularities of low body weight

The most prominent examples are headaches and migraines. Specifically, underweight individuals are considered to be belonging to the major group of patients with resistant and refractory migraine. To this end, lean patients have a specific muscle structural and functional patterns that participate in generating pain and provoke a specific muscle response patterns being more vulnerable to altered postural balance. Muscle and posture synergistically play a crucial role in particularities of the body composition relevant for increased pain perception and sensitivity that should be carefully considered for the individualised pain management in the lean patient cohort. Imaging data of muscle structure and function and overall posture is a rich source of biomarkers to be used for multi-level diagnostics, in order to stratify patients for treatments tailored to the person.

Myofascial trigger point (MTrP) is a pillar pathophysiological unit in development of myofascial pain and postural imbalance. To this end, dry needling of MTrP under ultrasound (US) guidance is a powerful approach to treat myofascial pain [72]. Migraine aetiology and course are strongly associated with myofascial pain, body mechanics and posture. Further to this, overall muscle structure can be significantly altered in body weight loss. Additionally to mechanical stimuli, cumulative signalling of hypoxic MTrPs and inadequate stress reactions synergistically can provoke migraine relapse. This effect is more pronounced in a lean body phenotype, due to specific characteristics such as overall body set-up and systemic effects including but not restricted to vascular dysregulation with consequent development of ischemic-hypoxic niches as well as shifted regulation of senses [4, 73].

Systemic alterations at the molecular level may further overlap between increased pain sensation/perception on the one hand and, on the other hand, specific reaction towards stress overload, inadequate temperature regulation and delayed and impaired healing processes with associated pathologies [9, 47]. To this end, more information is provided in the “Impaired wound healing” section below.

## Impaired wound healing

Impaired healing is a multifactorial health complication affecting a big portion of populations with tremendous socio-economic impacts worldwide. In the USA, around 6.5 million patients are affected with a dedicated budget of 25 billion US $ annually for treating chronic wounds [74]. Amongst the modifiable risk factors are unhealthy lifestyle, inappropriate nutritional habits, abnormal BMI and stress overload [24, 48].

Per evidence, underweight patients are at higher risk of mortality and poor outcome of general surgery compared to their overweight counterparts [49, 75]. Increased stress fracture rate and more severe course as well as delayed healing and return to sports activities were demonstrated for female runners with BMI ≤ 19 kg/m^2^ [76, 77]. Manrique et al. demonstrated a higher likelihood of surgical site infection and need for blood transfusion in the underweight total knee arthroplasty patients [49] potentially associated with impaired wound healing capabilities [49, 50]. Zusmanovich et al. demonstrated underweight as associated with increased length of a hospital stay [78]. Children’s Oncology Group reports on an increased risk of postoperative wound complication in low BMI patients with localised osteosarcoma; the authors recommended early nutritional intervention to reduce wound complications [79].

Strong association between low body weight and impaired wound healing was observed in breast cancer patients undergoing breast reconstruction surgery. Low BMI phenotype particularities such as disturbed microcirculation and altered pain perception were further associated with aggressiveness of the disease and surgical complications after breast surgery (Treskova I et al., unpublished data of the project-dedicated EPMA expert group).

## Cancers

A highly complex interrelation between individual body weight and cancer subtypes is a matter for a detailed evaluation, since individually abnormal body weight is an evident risk factor of and a contributor to the cancer development, progression and quality of individual outcomes [45, 80, 81]. Severe postoperative complications and poorer prognosis were associated with low BMI in a large cohort of gastric cancer patients undergoing gastrectomy, due to the lower haemoglobin and albumin, cachexia and compromised immunity. To this end, gastric cancer patients with higher BMI undergoing gastrectomy demonstrate significantly better survival rates compared to those with a BMI within the standard range [44]. Similarly, low BMI was demonstrated as an independent risk factor for increased mortality and co-incidence of cerebrovascular and pulmonary complications in resected lung cancer patients compared to their obese counterparts [82]. Underweight is suggested to be a predictor of poorer overall survival of colorectal cancer patients being potentially associated with poor performance status and decreased effectiveness of both the surgical and chemotherapeutic treatments [83]. Both obesity and underweight are related to a decreased survival in non-small-cell lung carcinoma (NSCLC) and small-cell lung carcinoma (SCLC) patients. A decrease in BMI from early adulthood to the time of diagnosis was associated with a 20 to 30% increase in poor survival of lung cancer patients [81].

Studies dedicated to prostate cancer–related mortality found out statistically significant risks associated with both high BMI (≥ 27.5 kg/m^2^) and low BMI (< 22.5 kg/m^2^) [84]. Further to this, data analysis performed towards 22 clinical trials revealed a positive association between overweight (BMI ≥ 25 kg/m^2^) and better overall survival amongst prostate cancer [85]. To this end, patient stratification based on the individualised patient profiling was highly recommended by the authors as being essential for the correct interpretation of the data, prediction and prognosis for the prostate cancer risks as the multifactorial disease [6].

Chen et al. demonstrated the underweight to be characteristic for breast cancer specifically in young Asian populations [45]. In consensus, underweight Korean premenopausal women were associated with an increased risk of breast cancer incidence [46]. Low body weight was associated with aggressive metastatic breast cancer also in the European clinical studies focused on symptoms and signs potentially associated with “pre-metastatic niches” [9, 47, 86]. In terms of currently run population screening programmes, underweight women receive less attention than overweight and obese women in the USA. These deficits have been finally recognised: promoting breast and cervical cancer screening amongst this currently underserved population is strongly recommended to reduce future disparities [87].

## Reproductive dysfunction and pregnancy

In 2019, young researchers Oksana Sergeeva and Vyktoria Kudryavtseva, Stavropol State Medical University, have been awarded in the category “Young professionals in 3P Medicine” by the European Association for Predictive, Preventive and Personalised Medicine for their discovery of the potential relevance of the vascular status of pregnant women with the FS phenotype for the foetal developmental particularities [88]. The authors reported that otherwise healthy mothers with some symptoms of the FS phenotype such as frequently cold extremities and characteristic low body weight, compared with the control group, demonstrate significantly increased vascular stiffness, altered gestational age (38.4 ± 0.81 vs. 39.5 ± 0.53) and trophic status of new-borns reflecting possible developmental particularities during the intrauterine period. To this end, in the target group, hypotrophy in new-borns was significantly more frequent compared to the controls (40% vs. 5.5%, respectively). Further to this, a significant difference was monitored for their peripheral augmentation index AIx75 in the first trimester compared to the controls [89]. Prior to pregnancy, initial body mass index in the target and control groups was reported as being 20.9 ± 1.1 kg/m^2^ and 23.2 ± 1.5 kg/m^2^, respectively. For mothers in the target group, such a focal infection as chronic tonsillitis was diagnosed 2.3 times more frequently compared to controls. Finally, signs of joint hypermobility (thumb symptom, wrist symptom etc.) as markers of the systemic connective tissue dysfunction were observed 3.2 times more frequently in pregnant and post-partum women of the target group compared to the controls, which in some cases was accompanied by changes in the valvular apparatus of the heart such as a mitral valve prolapse of varying severity (Sergeeva O, Evsevyeva M. et al., publication in preparation, 2021). More information about the association between connective tissue dysfunction and cardiovascular deficits characteristic for low body weight individuals is presented in the “Cardiovascular diseases” section.

There is a significant association between BMI and reproductive health [90]. Infertility problems and unfavourable outcomes in pregnancy are reported in women with low weight [53]. Anovulatory infertility was associated with underweight. Chronic deficiency of energy, strenuous exercise and stress overload can disrupt the hypothalamic-pituitary–gonadal (HPG) axis leading to the hypothalamic anovulation in young women [51]. Further to this, there is the risk of spontaneous abortion in underweight women. Non-classical congenital adrenal hyperplasia and ovarian dysfunction are prevalent in underweight women [90]. Although many women with non-classical congenital adrenal hyperplasia are generally fertile, there is a greater risk of subfertility, due to prevailing ovulatory dysfunction [52]. Low BMI was associated with negative effects also in vitro fertilisation (IVF) parameters such as decreased probability of pregnancy and increased risk of miscarriage. Further to this, for the FET IVF, Tang et al. demonstrated underweight as being associated with adverse pregnancy outcomes, reduced implantation rates and reduced rates of clinical pregnancy and ongoing pregnancy [91]. Low BMI is a risk factor of the male infertility, due to compromised semen quality [92].

## Compromised immunity

BMI and nutrition status are both highly relevant for an adequate immune response. As an extreme case, the primary immunodeficiency diseases (PID) are characterised as a group of congenital disabilities of the immunity that result in susceptibility to infections, autoimmunity, lymphoproliferation and atopy. A significantly higher prevalence of PID (adult and paediatric) is demonstrated in underweight with equal distribution of underweight patients in different PID diagnoses. Pre-existing poor nutritional status could modify the outcome of common variable immunodeficiency (CVID) predisposed individuals. Also, lymphopenia is observed in underweight children patients, while T cell lymphopenia is associated with CVID, lower immunoglobulin G levels and more autoimmune complications [56].

## COVID-19 and respiratory infections

There is a clear association between the individually physiologic body weight and the ability of an organism to defend against infectious diseases including COVID-19 [57, 93–95]. Obesity was associated with a more severe course of COVID-19. Further to this, Huang et al. recently demonstrate a J-shaped curve indicating that both underweight and obese patients have higher mortality when compared with patients with the normal body weight [93]. These results are supported by other investigations indicating more severe viral respiratory infections and respiratory mortality in underweight and obese patients evaluating influenza and other respiratory viruses [93–95]. BMI exerts a complex relationship with another respiratory infection—community-acquired pneumonia: underweight patients stay longer in the hospital. A prospective cohort study conducted on women from the Danish National Birth cohort showed an increased risk of community-acquired infectious diseases, including upper respiratory tract infections specifically for underweight individuals [96].

## Diagnostics of suboptimal low body weight

**Fig. 4:**
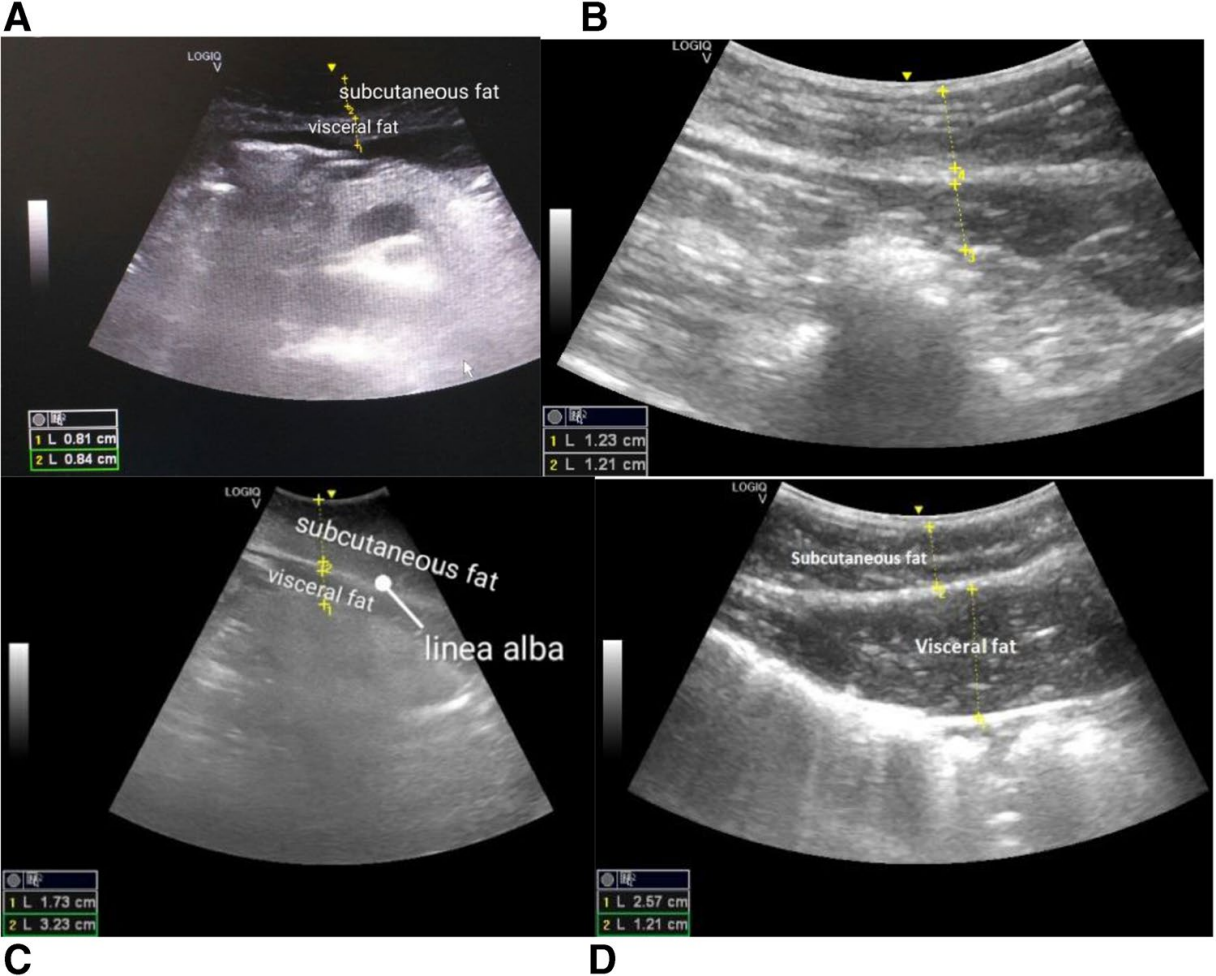
Ultrasound imaging as the diagnostic tool to discriminates between **A** abnormally low and **B** excessive versus **C** normal abdominal and visceral fat distribution and **D** SAT patterns in visceral fat redistribution; evident gender difference is well respected by gender-specific patterns of fat distribution, namely for males (**B** and **D**) and females (**A** and **C**). Notably, specific movement patterns at breathing further contribute to the correctness of the fat tissue measurement in abdominal cavity. The scanning was performed in the sagittal plane along the linea alba; the figure is adapted from [4]

As demonstrated in this position article, BMI is an important but certainly not decisive parameter for evaluation of the most optimal body shape individually. Furthermore, the standardised “normal” BMI might be misleading—the issue is well documented specifically for athletes and the elderly [97]. Individualised patient profiling utilising anthropometric data and medical and molecular imaging is instrumental to provide adequate diagnostics and personalised recommendations as presented earlier [4, 98, 99]. Medical imaging exemplified by evaluating subcutaneous (SAT) and visceral adipose tissue (VAT) measurements based on the ultrasound diagnostic approach is presented in Fig. 4. To this end, visceral versus subcutaneous types of the fat distribution demonstrate patterns specific for the patient stratification. Namely, subjects with *visceral* type of the fat distribution, rather than those with the *subcutaneous* type, may be significantly predisposed to an impaired flow-mediated endothelium-dependent vasodilatation [100, 101]. Relevant for both overweight and underweight individuals, strong correlations between the specific type of fat distribution and corresponding gene expression patterns have been demonstrated as a potential multi-level predictive diagnostic and prognostic tool [4, 102].

## Specific microbiome profiles codetermine suboptimal health of underweight individuals

Microbiome profiles are highly relevant for the periodontal and otolaryngologic health, digestive and urogenital tracts, healthy skin and physiologic wound healing, and mood disorders, amongst others [4, 6, 11, 13, 15, 103].

To this end, chronic inflammatory processes co-determined by shifted microbiome profiles frequently cause development and progression of severe pathologies such as cancers [23]. Further to this, xerostomia and vaginal dryness essentially associated with altered microbiome have been found to be more specific for young individuals demonstrating low body weight [11, 13].

Exemplified by an extreme condition under COVID-19 pandemic, it has been demonstrated that periodontopathic microflora is secondarily implicated in bacterial superinfection, systemic inflammation and pneumonia development, in severe cases leading to sepsis and death [103].

In consensus, over 50% of deaths in COVID-19 infected patients exhibiting bacterial superinfections and severe disease course were reported [41]. Microbiome profiles shifting towards Prevotella, Staphylococcus and Fusobacterium dominance representing periodontopathic bacteria were demonstrated for patients with poor COVID-19 outcomes.

Intestinal microbial population has been proven to a large extent to influence human health and maintaining homeostasis [104–107]. The setting of the gut microbiota depends on several factors such as host genetics, lifestyle, body activities, dietary habits, xenobiotics (e.g. antibiotics) and other drug intake as well as circadian rhythms [108–117]. In a number of studies, malnutrition and long-term dieting have been demonstrating as significantly influencing the gut microbiome and brain activity [118]. Remarkable alterations observed in animal models suggest that intestinal barrier dysfunction provoked by starvation may substantially contribute to the pathophysiology of anorexia nervosa [119]. To this end, also other eating disorders have been associated with a disturbed gut barrier function [120]. Pro-inflammatory cytokines levels significantly increased in acute anorexia nervosa are get further normalised by comprehensive nutritional rehabilitation [121, 122].

The “lean” microbiome is to a great extent unexplored issue considered by a very few scientific papers such as dedicated to the impaired wound healing in lean individuals [24, 48] and abundantly in social media that clearly demonstrates current discrepancy between pronounced interest in the population on the one hand but, on the other hand, very limited scientific efforts done to explore the topic.

## Nutritional mitigation of individual deficits

Nutritional aspects play a pivotal role in mitigating individual deficits potentially diagnosed in individuals with intentional and unintentional low body weight. Prominent examples linked to most frequently observed symptoms such as deficient microcirculation reflected in cold extremities, headaches, sleep deprivation, healing quality and pain severity are provided below.

### Deficient microcirculation reflected in cold extremities

There is a multifactorial causality of feeling cold and cold extremities of some individuals when other perceive temperature as comfortable; one of the reasons for that is a disturbed microcirculation frequently observed in vasospastic individuals e.g. with Flammer syndrome phenotype including low BMI [9]. Increased endothelin-1 levels and stress sensitivity synergistically lead to abnormal vasoconstriction reflected in characteristic cold extremities—the phenomenon which may be accompanied by cascading pathologies such as glaucomatous damage [7] and aggressive cancers [47, 86, 123].

Associated nutritional deficits are glucose, vitamin B, iron and magnesium amongst other dietary insufficiencies [4].

Increased levels of dietary magnesium may help to restore the balance between the blood levels of endothelin-1 as vasoconstrictor and NO as the vasodilator, therefore improving symptoms.

### Headaches

Fasting and hypoglycaemia are common triggers of headache and migraine in adults [124]. Affected individuals may be also very sensitive to one or more food components such as alcoholic drinks. The mechanism may be associated with the resulting dehydration and/or reaction towards ethanol, biogenic amines (including histamine, tyramine and phenylethylamine), sulphides, phenolic flavonoids, amongst others [124]. Further to this, micronutrient deficiencies such as vitamins B2 (riboflavin), B3 (niacin), B12 (cobalamin) and D and carnitine, α-lipoic acid and coenzyme Q10 have been associated with migraines; proposed mechanisms involve mitochondrial dysfunction, impaired antioxidant status and increased homocysteine levels [125]. Magnesium and nitrate supplementation is strongly recommended to restore vasodilatation and disturbed blood flow as the primary cause of headache and migraine in affected individuals [124, 126].

### Sleep deprivation

Despite a growing body of literature demonstrating an evident relationship between the high-quality workload performance and sleep, there is still a lack of awareness about the key role of sleep patterns in optimising individual performance. Clear evidence has been provided for creating appropriate strategies to optimise sleep patterns in elite athletes including expanding total sleep duration and quality, improving sleep environment and/or identifying potential sleep disorders [127].

Anorexia nervosa patients evidently suffer from reduced total sleep time and sleep onset latency. Duration and severity of malnutrition significantly influence their sleep quality, and at least partial weight restoration results in a “deepening” of nocturnal sleep in anorexic patients [128, 129].

Dieting and nutrients play a pivotal role in regulation of the sleep duration and quality, which in turn is decisive for prevention of many non-communicable diseases as well as poor outcomes under pandemic conditions e.g. in COVID-19 affected individuals [19]. To this end, high-carbohydrate diets and food containing tryptophan, melatonin and phytonutrients (e.g. cherries) have been demonstrated to improve sleep outcomes. The proposed mechanisms include serotonin and melatonin modulating pathways [130].

### Healing quality and pain severity

Evident risks predisposing an individual to an impaired healing and increased pain severity—both tightly linked together—are preventable in many cases resulting from suboptimal lifestyle and dietary habits, which in turn have been associated with risks linked to the abnormal body weight and suboptimal weight loss. To this end, specifically low BMI has been identified as the risk factor for complications, for example, in tissue expander surgery [131]. In a supervised weight loss programme, pain severity has been demonstrated as the predictor of suboptimal weight loss outcomes [132]. Modifiable risks and corresponding mitigating measures can get more evident by individualised patient profiling, utilising dedicated questionnaires and detailed molecular imaging [4, 25].

## Conclusions and expert recommendations in the framework of 3P medicine

### Standardised “normal” versus individually optimal body weight

An increasing interest in a healthy lifestyle raises questions about optimal body weight. Evidently, it should be clearly discriminated between *the standardised “normal” body weight* and *individually optimal weight*. To this end, the basic principle of personalised medicine “one size does not fit all” has to be applied. Contextually, “normal” (but e.g. borderline) BMI might be optimal for one person but apparently suboptimal for another one strongly depending on the individual genetic predisposition, geographic origin, cultural and nutritional habits and relevant lifestyle parameters—all included into comprehensive individual patient profile. Even if only slightly deviant, both overweight and underweight are acknowledged risk factors for a shifted metabolism which if being not optimised, in a long-term way, may strongly contribute to the development and progression of severe pathologies as highlighted above.

### Currently applied weight loss programmes raise questions and concerns

To this end, a follow-up study involving 20,002 adults was performed in the USA to analyse the efficacy of receiving weight-related advice from healthcare professionals. This study did not find that weight-related advice from healthcare professionals demonstrated any positive impact on BMI loss. Rather, opposite effects have been monitored. Namely, patients who reported receiving weight-related advice achieved worse weight outcomes a year later, compared to unsupervised patients. In-depth analysis of the issue and corresponding research programmes are essential to elucidate impacts of the weight-related recommendations, optimal lifestyle options and targeted prevention and treatment monitoring [133].

Occurrence of unexpected adverse effects following weight loss programmes may induce the accumulation of persistent organic pollutants into the bloodstream [134]. For example, intentional therapeutic loss of weight in diabetes type 2 patients potentially may not reach an expected decrease in risks to the disease on CVD, CVD mortality and all-cause mortality, due to methodological weaknesses [135]. Contextually, any effort in changing body weight has to be closely monitored by skilled healthcare professionals. The approach is comprehensive considering individualised patient profile and focused on the evidence-based benefits to the health condition of an individual in a long-term manner, therefore, following the principles of 3P medicine, namely application of predictive diagnostics, targeted prevention and treatment algorithms tailored to the person [136] and being safe, healthy, effective, nutritionally adequate and economically affordable and adapted to healthy individual and persons at risk [137]. If not, weight-modifying programmes might be useless or even harmful.

### Population health and innovative screening programmes

Development of innovative screening programmes is essential to promote population health by application of individualised patient profiling, multi-parametric analysis leading to cost-effective targeted prevention e.g. for individuals in suboptimal health conditions with a reversible damage [16, 71].

Amongst others, following healthcare areas are considered to strongly benefit from the above proposed measures:

- Sports medicine
- Suboptimal health conditions
- Stress overload and associated complications
- Planned pregnancies
- Periodontal health and dentistry
- Sleep medicine
- Eye health and disorders
- Inflammatory disorders
- Healing and pain management
- Metabolic disorders
- Cardiovascular disease
- Cancers
- Psychiatric and neurologic disorders
- Stroke of known and unknown aetiology
- Improved individual and population outcomes under pandemic conditions such as COVID-19.

In a long-term way, a significantly improved healthcare economy is one of the clear benefits of the proposed paradigm shift from reactive medicine to 3PM; a tight collaboration between all stakeholders including scientific community, healthcare providers, patient organisations, policy-makers and educators is essential for the smooth implementation of the 3PM concepts [136, 138–141].

Further issues linked to big data management and medical ethics have to be carefully treated in the context of application of artificial intelligence in medicine (below—see the “Application of artificial intelligence in medicine” section).

### Application of artificial intelligence in medicine

The advent of artificial intelligence (AI) and machine learning in the life science domain has opened immense opportunities to revolutionise the 3PMs. Leveraging the enormous amounts of biomedical data generated over the recent years (e.g. markers measured in biofluids, imaging modalities and various omics data), AI models can be built that learn from complex signals hidden in the data to make personalised predictions based on the biomarker signatures of individuals. Such models can, for example, predict clinical disease onset [11, 15–17, 71, 142], stratify patients into distinct sub-groups [10, 143–145], help understanding disease progression [71, 146] and aid as clinical decision support tools [147, 148].

AI methods are capable of integrating variables from various data modalities into large multivariate analyses which is especially important for highly complex, multifactorial diseases [149]. Here, the individual data types can lead to a more detailed discrimination between disease phenotypes and thus can improve the sensitivity and specificity when conducting personalised prognoses compared to univariate models.

Developing robust, trustworthy AI approaches is a challenging task that requires a deep understanding of the application domain, the available data and the technical approach [142, 146, 150]. This marks it a highly interdisciplinary endeavour where multiple experts need to collaborate. AI approaches catered towards the 3PMs depend on human data which is highly sensitive by nature. Therefore, a trade-off exists between ensuring data privacy through enforced regulations and enabling/accelerating research through open data sharing. It needs to be ensured that the AI models do not disclose any information about individuals, or discriminate against certain groups of humans due to sampling biases in the training data.

In the medical domain, AI approaches remained largely a research area until now rather than making the translation into clinical practice [151]. The reason for this is manifold: Before a proposed AI model can be applied in clinical practice, it needs to undergo several validation procedures to ensure accurate model performance and practical utility. First, the designed approach needs to be internally validated on the discovery dataset it was based upon, using appropriate internal validation procedures such as k-fold cross-validation. Second, the learned model needs to be retrospectively applied to external data (i.e. independent from the training data), where it should show approximately similar performance as on the original training data. Third, a prospective study has to be conducted for the model (similar to prospective studies for drug candidates), where model predictions are evaluated against the observations made in reality. Finally, regulatory institutions have to carefully decide whether application of the model is practically feasible, demonstrates a positive trade-off between risks and rewards and is considered ethical.

While there are ongoing challenges in the development and application of AI approaches in the biomedical context, there has been vast research progress over the last years. We believe that AI will stay a growing topic in the context of the 3PM over the upcoming years, potentially leading to an impactful paradigm shift with respect to how we think, pursue and enable the 3PM in research and clinical practice.

### Sports medicine 

Olympic games 2020/2021 in Japan refreshed professional discussions about the anorexia athletica and associated health risks observed in elite athletes such as the female athlete triad (disordered eating, amenorrhea, and osteoporosis) first described in 1992 by the American College of Sports Medicine and updated in 2007 including a spectrum of dysfunction related to energy availability, menstrual function, and bone mineral density. To this end, low energy supply, due to dietary restriction and increased expenditure, is considered to play a pivotal role in the triad development. Athletes involved in so-called “lean sports” which include but are not restricted to ballet, gymnastics and endurance running, are at highest risk. Still, there is an evident lack of effective screening programmes and management guidelines to mitigate health risks for the affected athletes. Effective risk assessment, targeted prevention and personalised intervention are critical to treat anorexia athletica. Multi-professional caregiver groups must remain vigilant in education, recognition, and treatment of athletes at risk [152].

## Data Availability

Not applicable.

## Abbreviations

ACE: Adverse childhood experiences
AF: Arterial fibrillation
AI: Artificial intelligence
BMC: Bone mineral content
BMI: Body mass index
CM: Childhood maltreatment
CT: Computer tomography
CVID: Common variable immunodeficiency
EPMA: European Association for Predictive, Preventive and Personalised Medicine
ET-1: Endothelin-1
EU: European Union
FET: Frozen-thawed embryo transfer
FFMI: Fat-free mass index
FS: Flammer syndrome
IVT: In vitro fertilisation
LACC: Locally advanced cervical cancer
MTrP: Myofascial trigger point
NSCLC: Non-small-cell lung carcinoma
PCa: Prostate cancer
PID: Primary immunodeficiency
ROS: Reactive oxygen species
SAT: Subcutaneous adipose tissue
SCLC: Small-cell lung carcinoma
UWL: Unintentional weight loss
VAT: Visceral adipose tissue
WHO: World Health Organization
3PM/PPPM: Predictive, Preventive Personalised Medicine
